# Clinical and Genetic Analysis of a European Cohort with Pericentral Retinitis Pigmentosa

**DOI:** 10.3390/ijms21010086

**Published:** 2019-12-20

**Authors:** Marianthi Karali, Francesco Testa, Raffaella Brunetti-Pierri, Valentina Di Iorio, Mariateresa Pizzo, Paolo Melillo, Maria Rosaria Barillari, Annalaura Torella, Francesco Musacchia, Luigi D’Angelo, Sandro Banfi, Francesca Simonelli

**Affiliations:** 1 Medical Genetics, Department of Precision Medicine, Università degli Studi della Campania ‘Luigi Vanvitelli’, via Luigi De Crecchio 7, 80138 Naples, Italy; karali@tigem.it (M.K.); annalaura.torella@unicampania.it (A.T.); 2 Telethon Institute of Genetics and Medicine, via Campi Flegrei 34, 80078 Pozzuoli, Italy; pizzomar@tigem.it (M.P.); f.musacchia@tigem.it (F.M.); 3 Eye Clinic, Multidisciplinary Department of Medical, Surgical and Dental Sciences, Università degli Studi della Campania ‘Luigi Vanvitelli’, via Pansini 5, 80131 Naples, Italy; francesco.testa@unicampania.it (F.T.); raffaella.brunettipierri@unicampania.it (R.B.-P.); valentina.diiorio@unicampania.it (V.D.I.); paolo.melillo@unicampania.it (P.M.); mariarosaria.barillari@unicampania.it (M.R.B.); luigi.dangelo@unicampania.it (L.D.A.)

**Keywords:** inherited retinal dystrophies, pericentral retinitis pigmentosa, next generation sequencing, *USH2A*

## Abstract

Retinitis pigmentosa (RP) is a clinically heterogenous disease that comprises a wide range of phenotypic and genetic subtypes. Pericentral RP is an atypical form of RP characterized by bone-spicule pigmentation and/or atrophy confined in the near mid-periphery of the retina. In contrast to classic RP, the far periphery is better preserved in pericentral RP. The aim of this study was to perform the first detailed clinical and genetic analysis of a cohort of European subjects with pericentral RP to determine the phenotypic features and the genetic bases of the disease. A total of 54 subjects from 48 independent families with pericentral RP, non-syndromic and syndromic, were evaluated through a full ophthalmological examination and underwent clinical exome or retinopathy gene panel sequencing. Disease-causative variants were identified in 22 of the 35 families (63%) in 10 different genes, four of which are also responsible for syndromic RP. Thirteen of the 34 likely pathogenic variants were novel. Intra-familiar variability was also observed. The current study confirms the mild phenotype of pericentral RP and extends the spectrum of genes associated with this condition.

## 1. Introduction

Retinitis pigmentosa (RP) defines a clinically heterogeneous group of inherited retinal dystrophies presenting with bilateral, progressive degeneration of photoreceptors (primarily rods) leading to night blindness, reduced-to-undetectable electroretinogram (ERG) amplitudes, peripheral field restriction, variable loss of central vision, and typical pigmentary degeneration of the retina [1]. The disorder has a prevalence of approximately 1 in 3000–4000 individuals with a total of more than one million affected people worldwide [1]. The clinical manifestation of RP is predominantly confined to the eye and the retina. However, approximately 20–30% of patients have extra-ocular involvement. Usher syndrome presenting with RP associated with hearing impairment is the most frequent condition among these syndromic cases [1].

Over 130 genes have been found to be responsible for RP (http://www.sph.uth.tmc.edu/RetNet; accessed on September 2019), with approximately 90 genes responsible for isolated, non-syndromic RP and about 40 genes for syndromic forms. The causative genes encode for proteins involved in a variety of fundamental processes that are responsible for the visual cycle, phototransduction, photoreceptor outer segment structure, and house-keeping functions such as transcription, RNA processing and ciliary function among others [2].

**Figure 1:**
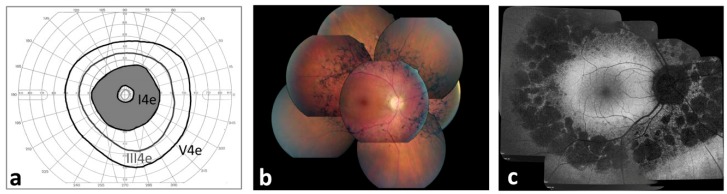
Ophthalmic features of pericentral RP. (**a**) Goldmann visual field of a representative pericentral RP patient showing the typical pericentral scotomas and preserved peripheral field. (**b**) Composite fundus photograph showing the typical retinal phenotype with sparing of far periphery. (**c**) Fundus autofluorescence (FAF) image illustrating hypo-autofluorescence within and beyond the vascular arcades.

Several RP subtypes have been clinically defined. Among them, pericentral RP is an atypical form with better preservation of the far periphery as a major distinctive feature. Specifically, subjects with pericentral RP display bone-spicule pigmentation or atrophy in the near mid-periphery while the far periphery, within and beyond the vascular arcades, is overall preserved leading to annular scotoma from 5–30 degrees (Figure 1). In these patients, full-field ERG responses are subnormal, yet still detectable [3].

Until now, the clinical features and genetic causes of pericentral RP have been reported only in non-European populations, by Comander et al. in 43 patients [4], Matsui et al. in 28 patients [5] and Sandberg et al. in 45 patients [3]. These reports described the clinical phenotypes in the different pericentral RP populations by monitoring the progress of the disease through the main morphological and functional parameters and compared the causative genes identified. Despite valuable insights on the genetic basis of this subtype of RP, precise genotype-phenotype correlations are yet to be firmly established.

In this study, we performed a detailed analysis of a large cohort of Italian patients with pericentral RP at the clinical and genetic levels. Our aim was to describe in depth the clinical features, to define the genetic causes by next-generation sequencing (NGS)-based procedures and to investigate possible genotype-phenotype correlations. To our knowledge, this is the first study of a European cohort of pericentral RP patients.

## 2. Results

### 2.1. Ophthalmological Characterization of Pericentral Retinitis Pigmentosa (RP) Patients

A total of 54 Italian patients from 48 independent families with pericentral RP at the time of presentation were recruited for the study. The majority of patients (*n* = 39) were sporadic cases with the exception of 15 families showing an autosomal recessive (*n* = 11) or an autosomal dominant (*n* = 4) pattern of inheritance (Table S1).

Subject mean age was 47.7 ± 18.9 years (range: 11–92 years) with a mean age of onset of 26.8 ± 17.8 years (range: 8 months–64 years) (Table 1, Table S1). Patients reported the following symptoms: night blindness (*n* = 26; 48.1%), reduction of visual acuity (*n* = 10; 18.5%), narrowing of visual field (*n* = 9; 16.7%) and photophobia (*n* = 6; 11.1%) (Table 1, Table S1). Only one patient (1.5%) presented nystagmus at the age of 8 months (Table S1). In 12 patients (22.2%) the diagnosis of RP was incidentally made (Table S1).

In terms of clinical manifestations, the pericentral RP cohort included patients diagnosed with isolated RP (*n* = 42) as well as patients presenting syndromic forms (*n* = 12) (Table S1). Specifically, 11 patients presented bilateral sensorineural hearing loss and were diagnosed with Usher syndrome type 2. Retrospectively, following the genetic analysis, one patient showing also amelogenesis imperfecta and nail abnormalities in addition to RP and hearing loss was diagnosed with Heimler syndrome (as explained below).

Mean best corrected visual acuity (BCVA) was 0.7 ± 0.3 decimals ranging from light perception to 20/20 in both eyes, with the vast majority of patients (*n* = 46; 85.2%) achieving a BCVA of 20/40 or better in at least one eye (Table S1). Refractive errors were on average −0.24 ± 2.37 diopters (D) and −0.15 ± 2.48 D in RE and LE, respectively, ranging from −4.00 D to 8.75 D (Table S1). The lens was clear in both eyes in 32 patients (59.3%), 12 patients (22.2%) showed initial lens opacity in both eyes, 6 patients (11.1%) showed subcapsular posterior opacity in both eyes, 3 patients (5.6%) were pseudophakic in both eyes, and 1 patient (1.9%) displayed subcapsular lens opacity in one eye and pseudophakia in the other eye (Table S1). The fundus of all patients was characterized by pigment accumulations within and beyond the vascular arcades while the far periphery had a normal appearance (Figure 1b).

The pericentral RP patients displayed different patterns of visual field loss. Specifically, we identified pericentral field loss in 43 eyes (43.9%), constricted with ring scotoma in 29 eyes (29.6%), constricted with peripheral scotoma in 9 eyes (9.2%), concentric constricted in 15 eyes (15.3%) and central scotoma in 2 eyes (2.0%). The mean Goldmann visual field examination (GVF) area using III4e target size was 3984.5 ± 2424.3^°2^ and 4041.6 ± 2497.3^°2^ in the right and left eyes, respectively. The mean GVF area using V4e target size was 7716.7 ± 3948^°2^ and 8332.3 ± 4486.7^°2^ in the right and left eyes, respectively (Table 1).

All patients underwent full-field ERG. Dark-adapted 0.01 responses (formerly called ‘rod response’) were below noise level in 15 patients (27.8%) and were reduced in 39 patients (72.2%) (mean b-wave amplitude: 28.7 ± 33.9 µV in RE; 28.2 ± 28.9 µV in LE; normal range: 95–305 µV) (Table S1). Dark-adapted 3.0 responses (formerly called ‘mixed response’) were subnormal (mean b-wave amplitude: 76.8 ± 70.9 µV and 80.9 ± 76.8 µV; normal range: 290–654 µV) in all analyzed patients (Table S1). Light-adapted 3.0 and 30 Hz were subnormal in all patients but one, who showed normal responses. Mean b-wave amplitude in light-adapted 3.0 were 36.3 ± 27.4 µV and 36.7 ± 26.6 µV (normal range: 103–250 µV) in the right and left eyes, respectively (Table S1). Mean P1-N1 amplitude in light-adapted 30 Hz responses were 23.9 ± 19.9 µV and 23.9 ± 19.4 µV (normal range: 57–223 µV) in the right and left eyes.

Optical coherence tomography (OCT) evaluation was performed in 50 pericentral RP patients (Table S1). Over half of the patients (55 eyes; 55.0%) showed vitreo-macular abnormalities (mean age: 46.4 ± 17.5 years). Forty-five eyes (45%) did not display any of the aforementioned macular alterations and had mean central retinal thickness (CRT) values at the range of 242.9 ± 47.3 µm, with reduced values in only 15 out of the 45 eyes (33.3%) (normal range: 270.2 ± 22.5 µm) (Table S1). Cystoid macular edemas (CME) were observed in 14 eyes (14.0%). Specifically, six patients showed bilateral CME and two patients showed CME only in one eye (Table S1). Epiretinal membranes (ERM) were observed in 15 eyes (15.0%): four patients had ERM bilaterally while nine patients showed ERM only in one eye (Table S1). One patient showed a bilateral lamellar macular hole (Table S1). The ellipsoid zone (EZ) band was measured in 46 patients (92.0%) with mean values of 2007 ± 1129 µm and 1964 ± 1116 µm in the right and left eyes, respectively (Table S1). For 4 patients (8.0%) the EZ band could not be assessed, because was absent, in both eyes.

For 24 patients (44.4%) fundus autofluorescence (FAF) imaging was performed (Table S1). Large areas of hypo-fluorescence were evident along the vascular course in all FAF analyses. Two patients (8.3%) had a normal macular autofluorescence in both eyes, whereas abnormal foveal hypo-autofluorescence was detected bilaterally in 4 patients (16.7%). The typical ring of perimacular hyper-autofluorescence was observed in 15 patients (62.5%). The rings were located at the posterior pole, inside vascular arcades, and its average width was 243 ± 236 µm in the right eyes and 243 ± 235 µm in the left eyes (Table S1). We also found a strong correlation between the width of the EZ band and the horizontal diameter of the external boundary of the hyper-autofluorescent ring (ρ > 0.9; *p*-value < 0.001). Finally, a hyper-autofluorescent foveal patch was seen in 3 patients (12.5%).

Nineteen patients (35.2%) performed microperimetry (MP1) (Table S1). Mean retinal sensitivity was 5.5 ± 5.8 dB and 4.7 ± 5.1 dB in the left and right eyes, respectively. The fixation was stable in 22 out of the 38 eyes (57.9%), relatively stable in 11 eyes (28.9%) and unstable in 5 eyes (13.2%) (Table S1). Nine patients showed a stable fixation in both eyes, while fixation was relatively stable in 4 patients and unstable in 2 patients.

### 2.2. Genetic Analysis

A total of 39 patients (from 35 unrelated families), clinically classified as pericentral RP, gave their informed consent to genetic testing. Genomic DNA was analyzed by targeted NGS using either panel-based sequencing of known retinopathy genes or a more comprehensive clinical exome sequencing aiming at sequencing more than 2700 genes responsible for Mendelian disorders.

**Figure 2:**
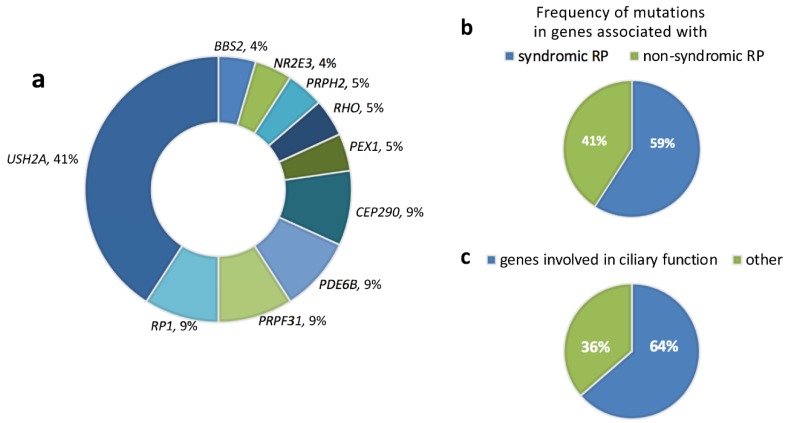
Frequency of causative genes in the pericentral RP cohort. (**a**) Relative frequency of retinopathy genes mutated in the 23 familial cases with pericentral RP. (**b**,**c**) Prevalence for causative variants in genes associated also with syndromic retinopathies and in genes involved in ciliary function.

Causative mutations were found in 22 out of 35 analyzed families (63% diagnostic rate). Pathogenic or likely pathogenic variants were identified in the following genes: *USH2A* in 9 families (representing 41% of solved cases and 25.7% of total cases); *CEP290* in 2 families (9%; 5.7%); *RP1* in 2 families (9%); *PRPF31* in 2 families (9%; 5.7%); *PDE6B* in 2 families (9%; 5.7%); *BBS2* in 1 family (4.5%; 2.8%); *NR2E3* in 1 family (4.5%; 2.8%); *PEX1* in 1 family (4.5%; 2.8%); *PRPH2* in 1 family (4.5%; 2.8%); *RHO* in 1 family (4.5%; 2.8%) (Table 2; Figure 2a). Pathogenic variants were validated by Sanger sequencing. Segregation analysis was performed whenever possible, confirming the expected zygosity state and the presence of the pathogenic variants in *trans*.

Thirteen of the 34 putatively pathogenic variants identified in this study (Table 2) have not been previously described, as assessed by cross-checking the entries in the Human Gene Mutation Database (HGMD) or the Leiden Open Variation Database (LOVD) (Table 3). The novel variants included one nonsense variant in *RP1* (c.2219C > G), two frameshift variants in *RP1* (c.2978delC) and *PRPF31* (c.690delG), two splicing variants in *CEP290* (c.5709 + 2T > G) and in *USH2A* (c.5776 + 1G > C), a non-frameshift insertion in *PEX1* (c.2145_2146insTCTCAG) and seven missense variants (in *CEP290*, *PRPF31*, *RHO*, and *USH2A*) (Table 3). The pathogenicity of the missense variants was corroborated by in silico predictions of their impact on protein function (Table 3) and by segregation analysis, whenever possible.

The aforementioned genetic analysis allowed us to detect a high frequency of pathogenic variants in genes that are usually associated with syndromic RP (Figure 2b). Collectively, 59% of the patients with pericentral RP harbored mutations in *BBS2*, *CEP290*, and *USH2A* which, besides non-syndromic forms, are responsible for Bardet-Biedl syndrome (www.omim.org/entry/606151), Joubert, Meckel and Senior-Loken syndromes (www.omim.org/entry/610142), and Usher syndrome type 2 (www.omim.org/entry/608400), respectively. Interestingly, one of the analyzed patients (P27) was found to harbor two putatively pathogenic variants (c.2145_2146insTCTCAG and c.274G > C; in *trans)* in the *PEX1* gene, which is responsible for a perixosome biogenesis disorder (i.e., Heimler syndrome) (www.omim.org/entry/602136). Indeed, a more careful clinical re-evaluation of P27 revealed the presence of signs compatible with Heimler syndrome, such as dental anomalies and sensorineural hearing loss (Barillari et al., manuscript in preparation).

*USH2A*, which is the most common genetic cause of Usher syndrome, was the most frequently mutated gene in our cohort, accounting for over one third of the cases analyzed (9 out of the 25 solved familial cases). Eight patients (7 families) harboring mutations in *USH2A* displayed pericentral RP in the context of an Usher syndrome type II, whereas the other two patients with causative variants in *USH2A* showed a normal audio-vestibular response.

Intriguingly, almost two thirds of the solved cases of pericentral RP (i.e. 14 out of 22) harbored putatively pathogenic mutations in genes involved in ciliary development and function, such as *BBS2* (one case), *CEP290* (two cases), *RP1* (two cases) and *USH2A* (nine cases) (Figure 2c).

### 2.3. Intrafamilial Variability of Pericentral RP

The ophthalmological analysis of our pericentral RP cohort revealed marked intrafamilial phenotypic variability in at least three cases.

**Figure 3:**
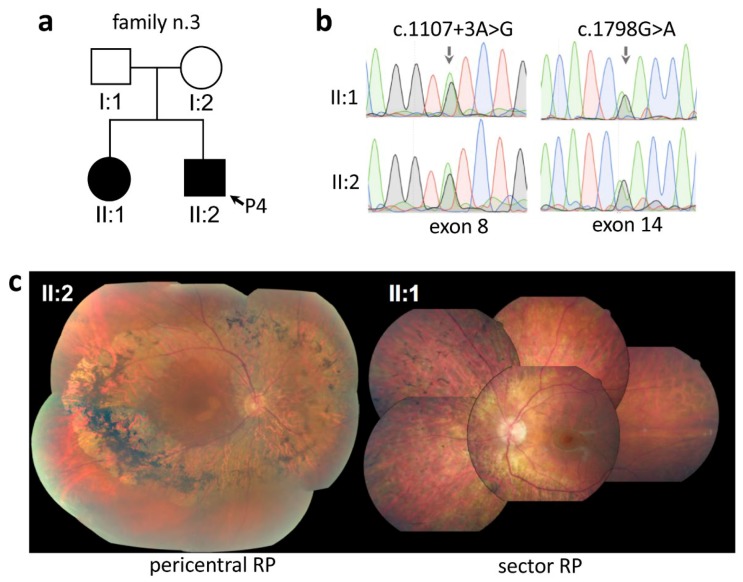
Example of intrafamiliar phenotypic variability. (**a**) Pedigree of family n. 3. (**b**) Sanger sequencing traces of the two variants in exon 8 and exon 14 of the *PDE6B* gene in the two affected siblings (II:1, II:2) of family n. 3. (**c**) Composite fundus photograph of the proband (II:2, P4) and his 6 years older affected sister (II:1), revealing typical features of pericentral RP (left hand side) and sector RP (right hand side), respectively.

In the case of family n. 3, both offspring of unaffected parents were diagnosed with RP (Figure 3a). One of the siblings (II:2) was diagnosed with pericentral RP and was included in our cohort (P4; Table 1). This patient, at the age of 49, displayed as first symptoms a reduction of visual field, preserved BCVA (20/23 in both eyes), the typical pericentral retinal phenotype at the fundus examinations (II:2 in Figure 3c), constricted visual field with ring scotoma, and subnormal dark-adapted 0.01 and light-adapted ERG responses (Table S1). His affected sister, during a routine ophthalmological visit at the age of 57, showed a sector form of RP (i.e., bone-spicule localized in the nasal sector)(II:1 in Figure 3c) with preserved visual acuity (20/20 in both eyes), slightly constricted visual field, dark-adapted 0.01 ERG response below noise level, and subnormal light-adapted ERG responses. Both affected siblings were found to carry compound heterozygous pathogenic variants (c.1107 + 3A > G and c.1798G > A) in the *PDE6B* gene (Table 2, Figure 3b).

We observed phenotypic variability also in family n. 25 between patient P29 (Table 1) and her affected brother. The proband (P29) showed typical pericentral RP findings with incidental diagnosis at the age of 9, BCVA of 20/200 in right eye and 20/30 in left eye, the typical pericentral retinal phenotype at the fundus examination, pericentral visual field loss, subnormal dark-adapted 0.01 and light-adapted ERG responses (Table S1). Instead, her brother was diagnosed at 20 years with typical RP, characterized by a severely restricted visual field (10° using target size III4e) and ERG responses below noise level. Molecular analysis revealed that both affected siblings were compound heterozygous for two likely pathogenic variants (c.3045C > G and c.6992G > A) in the *USH2A* gene (Table 2). Segregation analysis in the family suggested that the variants were in *trans*. Hearing tests did not reveal any audio-vestibular alterations in either patient.

Finally, in family n. 43, the daughter of patient P49 (Table 2), during a routine ophthalmological visit at the age of 25 years, showed a sector form of RP (i.e., bone-spicule localized in the temporal sector), with ERG responses at the lower limit of the normal range and a slightly reduced visual field. On the other hand, P49 displayed a disease onset at the age of 42 with night blindness and reduction of visual field, preserved BCVA (20/20 in both eyes), the typical retinal phenotype at the fundus examination, pericentral visual field loss, subnormal dark-adapted 0.01 and light-adapted ERG responses (Table S1). Genetic analysis was performed in both patients but no convincing pathogenic variants were identified.

## 3. Discussion

In the present study, we provide a comprehensive clinical and genetic evaluation of subjects with pericentral RP. To our knowledge, this study includes the largest cohort of subjects (54 cases from 48 unrelated families) of European origin with pericentral RP.

Consistent with previous reports [21], we found that pericentral RP is a rather uncommon RP subtype and has extensive genetic heterogeneity. Genetic defects underlying pericentral RP are overall overlapping those causing mild forms of typical RP, as previously suggested [4]. While previous studies [3,4,5] found pericentral RP cases almost exclusively in the context of non-syndromic forms, we observed a high prevalence of pericentral RP within the syndromic RP group, mainly in Usher syndrome. In agreement, variants in *USH2A* were detected in over 40% of the solved cases in our cohort. A high frequency of *USH2A* mutations among cases with pericentral RP has not been previously reported [4,5,22,23]. Different studies identified *RHO* [4,24] or *ABCA4* [5] defects as the most common cause. The inclusion criteria for each study cohort may partly account for this difference. Specifically, Matsui et al. included only subjects with non-syndromic retinal degenerations, while Grondahl et al. analyzed only four genes (*RHO*, *RP1*, *PRPH2* and *IMPDH1*) that did not comprise *USH2A*. Our cohort can be directly compared to the study of Comander et al. which recruited 45 cases with pericentral RP without excluding syndromic forms [4]. In that report, *USH2A* gene defects were identified in 14.3% of solved cases, whereas mutations in the *HGSNAT* gene (typically responsible for Mucopolysaccharidosis type IIIC [25]) were a relatively common cause of pericentral RP [4]. However, the cohorts’ size (i.e., 54 and 45 cases) does not allow to discuss further the discrepancies in mutation frequency. An expansion of the pericentral RP cohorts analyzed is, therefore, necessary to draw firm conclusions on any significant enrichment in specific gene contributions to the pathogenesis of this condition.

In the present study, among the 10 familial cases with mutations in *USH2A*, eight had an associated sensorineural hearing loss and were diagnosed as Usher syndrome type 2. This subgroup of patients with mutations in *USH2A* displayed a milder phenotype compared to a previously described cohort of syndromic and non-syndromic RP patients [26]. In particular, BCVA was better than 20/40 in at least one eye in our pericentral RP patients with *USH2A* variants (average age: 50 years), whereas a large group of 105 patients with *USH2A* variants (mean age: 32 years; i.e., almost 20 years younger than our pericentral RP subgroup) showed a BCVA higher than 20/40 in 83% of subjects [26]. Moreover, ERG response was more preserved, e.g., 30 Hz ERG in our *USH2A* pericentral RP patients (22 µV on average), while Sandberg et al. reported a mean amplitude of 6 µV in *USH2A* patients [26].

Traditionally, RP has been studied through the main morphological and functional parameters such as visual acuity, GVF, and ERG. For this reason, we compared these measurements from our cohort of pericentral RP patients to those from patients with typical RP as well as from other cohorts of pericentral RP described in literature. Pericentral RP was found to be associated with a better visual acuity compared to typical RP. Indeed, almost all patients (85.2%) showed a preserved BCVA of 20/40 or better in at least one eye, in line with the previous studies on pericentral RP that reported a percentage of 71% and 94% [3,4,5]. In particular, in a large US cohort of typical RP (about 900 cases), only 55% of subjects had a visual acuity of 20/40 or better [27]. This percentage is even lower when considering older subgroups. For instance, Grover et al. [28] reported that only 52% of typical RP patients aged ≥45 years had a visual acuity of 20/40 or better (84% in our cases with ≥45 years). Similarly, Berson et al. [29] reported that 54% of RP patients aged ≥40 years had a visual acuity of 20/40 or better (85% in our cases aged ≥40 years). Furthermore, the GVF area estimated in pericentral RP was wider compared to typical RP, in agreement with the above-cited studies [3,4,5]. In particular, the GVF area measured with V4e target appeared markedly more preserved in pericentral RP patients compared to a large cohort (*n* = 601) of typical RP patients recruited in a clinical trial investigating the effects of vitamin A treatment in RP [30]. The baseline of this study reported an average area of about 2.000^°2^ [30]. This difference is significant taking into consideration that subjects of an older age (>50 years) and with more compromised visual function (i.e., VA <20/200, GVF diameter <8°, non-recordable ERG) were excluded from the latter study [30]. Finally, 30 Hz ERG were more preserved in pericentral RP patients (average: 24 µV) compared to typical RP patients (average: 7 µV in subjects with mutations in *RHO* [24]; 6 µV in subjects with mutations in *USH2A* [26]; 2 µV in subjects with mutations in *RPGR* [31]). Taken together, these features underline a better clinical course of pericentral RP compared to typical RP in agreement with the other studies on patients with pericentral RP [3,4,5,24,31].

The OCT-based assessment of macular abnormalities revealed that the frequency of CME in pericentral RP patients (i.e., 16.0%) was comparable with that reported in our cohort of typical non-syndromic RP (22.9%) and Usher syndrome (15.7%). In contrast, the frequency of ERM (15%) was slightly lower in the pericentral RP cohort (typical non-syndromic RP: 19.8%; Usher syndrome: 19.0%). The other OCT findings (e.g., CRT and EZ width) were comparable with those in typical RP cohorts. FAF imaging revealed a pattern of hyper-autofluorescent ring in most subjects, similar to that observed in typical RP [32,33].

Regarding intrafamilial variability, also Comander et al. [4] described a case of two brothers harboring the same mutation in *PRPF31* but presenting different retinal phenotypes. One child showed a typical RP while the other displayed all the features of pericentral RP.

Mutations in the *CEP290* gene are reported as the most common (20%) cause of Leber congenital amaurosis (LCA) and they are more frequently associated with severe forms of retinopathies [34,35]. In this study, we found two subjects (P10, P24) with mutations in *CEP290* among the cases with pericentral RP. P10 had a disease onset at the age of 5 years with reduction of visual field, preserved BCVA (20/20 in both eyes), constricted visual field with ring scotoma, subnormal dark-adapted 0.01 and light-adapted ERG responses (Table 1, Table S1). Patient P10 was found to be compound heterozygous for two pathogenic variants in *CEP290* (c.5709 + 2T > G and c.384_385del) (Table 2). P24 displayed an incidental diagnosis at the age of 12 years, preserved BCVA (20/20 in both eyes), peripheral visual field loss in the right eye and costricted visual field with ring scotoma in the left eye, subnormal dark-adapted 0.01 and light-adapted ERG responses (Table 1, Table S1). P24 was found to be compound heterozygous for two missense variants in *CEP290* (c.1664A > T and c.1092T > G) (Table 2). To our knowledge, these are the first reported cases of *CEP290* defects associated with pericentral RP.

The current study has some limitations related to its retrospective and cross-sectional design. In particular, a single visit is available for most subjects in contrast to the study of Comander et al. [4] in which 11 patients were evaluated on multiple visits. Moreover, some ophthalmological tests (i.e., OCT, FAF, MP) were not performed in all subjects. Nevertheless, our study, carried out for the first time on patients of European origin, adds important insights into the clinical presentation and genetic defects of pericentral RP. Interestingly, it expands the number of genes associated with the pericentral RP phenotype, including those involved in Usher and other syndromic conditions. In light of this finding, we suggest that whenever a pericentral RP is diagnosed, it is important to perform audio-vestibular examination, even in the absence of hearing symptoms, to unravel a potentially masked Usher syndrome. Moreover, the clinical characterization of our cohort confirms the mild phenotype of pericentral RP, particularly in terms of visual acuity, visual field and ERG responses. Finally, it highlights the high degree of intrafamilial phenotypic variability, even among siblings of a comparable age, suggesting that both genetic and environmental factors may act as disease modifiers.

## 4. Materials and Methods

### 4.1. Ethics Statement

All procedures were approved by the Ethics Boards of the University of Campania ‘Luigi Vanvitelli’ (protocol n. 0008189/2015, 09.04.2015) and adhered to the tenets of the Declaration of Helsinki. Samples were collected upon written informed consent of the patient to sample collection and genetic analysis. For minors, informed consent was obtained by the parents or legal guardians.

### 4.2. Patients Inclusion Criteria

The medical records of the patients were taken at the Referral Center for Inherited Retinopathies of the Eye Clinic of the University of Campania ‘Luigi Vanvitelli’ from November 2006 to June 2019. The patients were enrolled for the study on the basis of their retinal phenotype. The inclusion criteria were: bone-spicule pigmentation or atrophy in the near mid-periphery (5–30 degrees) within and beyond the vascular arcades, corresponding to an annular scotoma, normal appearance of optic disk macula and far retinal periphery (Figure 1); and subnormal, yet recordable, full-field ERG responses. Patients with a medical history of hydroxychloroquine use were excluded, since the toxicity induced by hydroxychloroquine (Plaquenil) has been reported as a non-genetic cause of the pericentral RP phenotype [36].

To establish the onset of the disease, patients were questioned about the age at which they first noticed night vision problems and visual disturbances. The duration of the disease was calculated over time between the onset of symptoms and the date of the examination. Family history was also collected at the first visit. In case of incidental diagnosis, the age at diagnosis was considered as the disease onset.

Pedigree information was used to determine patterns of inheritance. RP was categorized as autosomal dominant (AD) (e.g., one affected parent and child, equal gender distribution), autosomal recessive (AR) (e.g., no affected parents, consanguinity) and sporadic (SP) cases (i.e., patients with no affected relatives).

### 4.3. Ophthalmological Examination

All patients underwent a full ophthalmological examination which included: best-corrected visual acuities (BCVA) measured using the Snellen chart, slit lamp anterior segment examination, fundus examination, Goldmann visual field (GVF) examination, ERG, microperimetry (MP1), optical coherence tomography (OCT) and fundus autofluorescence (FAF) imaging.

GVF was measured in 49 patients by moving the III4e and V4e stimulus target on a calibrated standard Goldmann perimeter by the same experienced ophthalmic technician and was analyzed as previously published [37].

Full-field ERG was recorded by corneal contact lens electrodes with a Ganzfeld stimulator (EREV 2000 Electrophysiology system; LACE Elettronica, Pisa, Italy) according to the recommendations of the International Society for Clinical Electrophysiology of Vision (ISCEV) [37].

MP1 was performed in 19 patients by an automatic fundus-related perimeter (MP1 Microperimeter, Nidek Technologies, Padova, Italy). The following parameters were used: a fixation target of 2° in diameter consisting of a red ring; a white, monochromatic background with a luminance of 1.27 cd/m^2^; a Goldmann III-size stimulus with a projection time of 200 ms; and predefined automatic test pattern (Humphrey 10-2) covering 10° centred onto the gravitational centre of the fixation points with 68 stimuli.

OCT was performed in 50 patients with the spectral domain OCT (SD-OCT) (Cirrus HD-OCT, Carl Zeiss, Dublin, CA, USA) by an experienced operator. The acquisition protocol comprised both a five-line raster scan and a macular cube scan pattern (512 × 128 pixels) in which a 6 × 6 mm region of the retina was scanned within a scan time of 2.4 seconds.

FAF imaging was performed bilaterally in 24 patients according to the protocol reported by Aboshiha et al. [38]. Briefly, fundus autofluorescence imaging of 30° field of view and 8 mm was acquired. Measurements of structures on FAF were performed using the Spectralis software with micrometer caliper. Temporal horizontal radius of the hyperautofluorescent ring (distance from fovea to inner and outer border) or patch was measured on the FAF image [39]. FAF were classified following the patterns proposed by Fakin et al. [39], i.e, hyperautofluorescent ring, hyperautofluorescent foveal patch and abnormal central hypoautofluorescence.

The patients referring extraocular abnormalities or bearing mutations in genes associated with syndromic conditions, whenever the clinical observation could not firmly exclude a syndromic form, underwent the appropriate specialist control (e.g., audiological visits for patients reporting hearing problems or harboring mutations in *USH2A*; dental and dermatological visits for patients harboring mutations in *PEX1*, etc.).

### 4.4. Next-Generation Sequencing

Genomic DNA was extracted from peripheral blood using the DNeasy Blood and Tissue Kit (QIAGEN) according to the manufacturer’s instructions. Samples were analyzed either by panel-based sequencing of known retinopathy-associated genes or by clinical exome sequencing using the ClearSeq Inherited Disease panel (Agilent). Libraries were sequenced on a NextSeq 500 sequencing platform (Illumina inc., San Diego, CA, USA) in collaboration with the NGS Core at the Telethon Institute of Genetics and Medicine (TIGEM).

### 4.5. Variant Analysis and Interpretation

Sequencing data were analyzed in collaboration with the Bioinformatics Core at TIGEM using a previously described in-house developed pipeline [40]. The single nucleotide variants (SNVs) and indel variants were annotated as described [40,41]. The alignments at candidate positions were visually inspected using the Integrative Genomics Viewer (IGV). To assess the possible deleterious effects of missense variants, in silico predictions of pathogenicity from the MutationTaster (http://www.mutationtaster.org/) [42], Polymorphism Phenotyping v2 (Polyphen-2; http://genetics.bwh.harvard.edu/pph2/) [43], Sorting Intolerant from Tolerant (SIFT; http://sift.bii.a-star.edu.sg/) [44] and Combined Annotation-Dependent Depletion (CADD; http://cadd.gs.washington.edu/) [45] tools were considered.

### 4.6. Variant Validation

The identified variants were validated by Sanger sequencing of the corresponding genomic fragments. For the amplification of the exon sequence, polymerase chain reaction (PCR) was performed on 20 ng of genomic DNA using Taq polymerase according to standard protocols. Amplicons were Sanger sequenced and mutation detection was performed using the CodonCode Aligner software. Segregation analysis was performed for the majority of patients (with only a few exceptions when parents’ blood samples were not available).

## Figures and Tables

**Table 1:** Main clinical findings in the patients with pericentral retinitis pigmentosa (RP).

Family	Gene Mutation	Patient	Age of Onset (yrs)	Age (yrs)	BCVA RE	BCVA LE	V4e Field Description RE	V4e Field Description LE
F1	*PRPF31*	P1	8	15	1	1	pericentral field loss	pericentral field loss
		P2	12	49	1	1	constricted with peripheral scotoma	constricted with peripheral scotoma
F2		P3	30	55	0.8	1	constricted with ring scotoma	constricted with ring scotoma
F3	*PDE6B*	P4	49	52	0.9	0.9	constricted with ring scotoma	constricted with ring scotoma
F4	*BBS2*	P5	15	20	1	1	pericentral field loss	pericentral field loss
F5		P6	50	84	0.2	0.4	n.a.	n.a.
F6		P7	31	51	1	1	pericentral field loss	pericentral field loss
		P8	25	55	0.7	0.9	constricted with ring scotoma	constricted with ring scotoma
F7	*RP1*	P9	47	52	1	1	constricted with ring scotoma	constricted with ring scotoma
F8	*USH2A*	P10	11	47	1	1	constricted with ring scotoma	constricted with ring scotoma
		P11	38	58	0.7	0.6	constricted with ring scotoma	constricted with ring scotoma
F9	*USH2A*	P12	14	40	0.8	0.9	concentric constricted	concentric constricted
F10	*CEP290*	P13	5	34	1	1	constricted with ring scotoma	constricted with ring scotoma
F11	*USH2A*	P14	20	44	0.9	0.9	constricted with peripheral scotoma	constricted with peripheral scotoma
F12	*PRPF31*	P15	4	22	1	1	pericentral field loss	pericentral field loss
		P16	5	27	1	1	constricted with peripheral scotoma	concentric constricted
F13	*USH2A*	P17	11	44	0.7	0.5	concentric constricted	concentric constricted
F14	*PRPH2*	P18	27	45	1	0.8	constricted with ring scotoma	constricted with ring scotoma
F15		P19	57	76	0.0016	0.0016	n.a.	n.a.
F16		P20	47	59	1	1	constricted with ring scotoma	constricted with ring scotoma
F17		P21	9	27	0.1	0.1	pericentral field loss	pericentral field loss
F18		P22	36	47	0.8	1	constricted with ring scotoma	constricted with ring scotoma
F19		P23	30	53	1	0.9	constricted with peripheral scotoma	constricted with peripheral scotoma
F20	*CEP290*	P24	12	19	1	1	pericentral field loss	constricted with peripheral scotoma
F21		P25	41	43	1	1	pericentral field loss	pericentral field loss
F22	*RHO*	P26	9	14	1	1	pericentral field loss	pericentral field loss
F23	*PEX1*	P27	10	11	0.3	0.3	pericentral field loss	pericentral field loss
F24		P28	34	50	0.2	0.1	n.a.	n.a.
F25	*USH2A*	P29	43	49	0.1	0.7	n.a.	n.a.
F26	*USH2A*	P30	15	56	0.8	0.4	pericentral field loss	pericentral field loss
F27		P31	53	59	0.6	1	constricted with ring scotoma	constricted with ring scotoma
F28	*USH2A*	P32	42	48	0.7	0.8	pericentral field loss	pericentral field loss
F29		P33	13	20	0.9	0.6	pericentral field loss	pericentral field loss
F30		P34	5	58	0.8	0.8	concentric constricted	concentric constricted
F31	*PDE6B*	P35	19	41	0.8	1	constricted with ring scotoma	constricted with ring scotoma
		P36	15	43	0.05	0.05	constricted with ring scotoma	constricted with ring scotoma
F32	*NR2E3*	P37	0.67	22	0.6	0.9	pericentral field loss	pericentral field loss
		P38	3	27	0.6	0.6	pericentral field loss	pericentral field loss
F33	*USH2A*	P39	40	69	0.6	0.8	central scotoma	central scotoma
F34		P40	49	58	0.7	0.9	pericentral field loss	pericentral field loss
F35		P41	45	64	0.6	0.5	concentric constricted	concentric constricted
F36		P42	5	40	0.7	0.7	pericentral field loss	pericentral field loss
F37		P43	50	78	0.1	0.05	concentric constricted	concentric constricted
F38		P44	55	60	0.9	1	pericentral field loss	pericentral field loss
F39		P45	27	71	1	0.4	n.a.	n.a.
F40	*RP1*	P46	38	42	0.9	0.6	pericentral field loss	pericentral field loss
F41	*USH2A*	P47	24	45	0.6	0.6	concentric constricted	concentric constricted
F42		P48	38	44	1	0.9	constricted with ring scotoma	constricted with ring scotoma
F43		P49	14	68	1	1	pericentral field loss	pericentral field loss
F44		P50	64	80	0.8	0.8	concentric constricted	concentric constricted
F45		P51	6	92	0.3	0.4	constricted with peripheral scotoma	constricted with ring scotoma
F46		P52	50	77	0.6	0.5	pericentral field loss	pericentral field loss
F47		P53	18	39	0.8	0.7	pericentral field loss	pericentral field loss
F48		P54	15	33	0.9	0.9	pericentral field loss	pericentral field loss

Abbreviations: BCVA, Best-corrected visual acuity; LE, Left eye; n.a., not available; RE, Right eye.

**Table 2:** Genetic findings in the patients with pericentral RP.

Patient	Family	Gene	RefSeq	Allele 1				Allele 2		
				Nucleotide	Protein	Reference		Nucleotide	Protein	Reference
*P1*	F1	*PRPF31*	NM_015629	c.3G > A	p.(Met1Ile)	this study		-	-	-
*P4*	F3	*PDE6B*	NM_000283	c.1107 + 3A > G	p.(?)	[6]		c.1798G > A	p.(Asp600Asn)	[7]
*P5*	F4	*BBS2*	NM_031885.3	c.401C > G	p.(Pro134Arg)	[8]		c.401C > G	p.(Pro134Arg)	[8]
*P9*	F7	*RP1*	NM_006269	c.2219C > G	p.(Ser740 * )	this study		-	-	-
*P46*	F40	*RP1*	NM_006269	c.2978delC	p.(Ser993Phefs * 20)	this study		-	-	-
*P10*	F8	*USH2A*	NM_206933	c.497A > G	p.(Glu166Gly)	this study		c.14977_14978del	p.(Phe4993Profs * 7)	[9]
*P12*	F9	*USH2A*	NM_206933	c.9815C > T	p.(Pro3272Leu)	[10]		c.949C > A	p.(Arg317Arg)	[11]
*P13*	F10	*CEP290*	NM_025114	c.5709 + 2T > G	p.(?)	this study		c.384_385del	p.(Asp128Glufs * 17)	[12]
*P14*	F11	*USH2A*	NM_206933	c.4732C > T	p.(Arg1578Cys)	[9]		c.14885dup	p.(Glu4963Glyfs * 38)	[13]
*P15*	F12	*PRPF31*	NM_015629	c.690delG	p.(Ile231Serfs*8)	this study		-	-	-
*P17*	F13	*USH2A*	NM_206933	c.4717C > T	p.(Gln1573*)	[13]		c.10712C > T	p.(Thr3571Met)	[14]
*P18*	F14	*PRPH2*	NM_000322	c.458A > G	p.(Lys153Arg)	LOVD ^‡^		-	-	-
*P24*	F20	*CEP290*	NM_025114	c.1664A > T	p.(Lys555Ile)	this study		c.1092T > G	p.(Ile364Met)	this study
*P26*	F22	*RHO*	NM_000539	c.560G > T	p.(Cys187Phe)	this study		-	-	-
*P27*	F23	*PEX1*	NM_000466	c.274G > C	p.(Val92Leu)	[15]		c.2145_2146insTCTCAG	p.(Gln716delinsSerGlnGln)	this study
*P29*	F25	*USH2A*	NM_206933	c.3045C > G	p.(His1015Gln)	this study		c.6992G > A	p.(Gly2331Glu)	this study
*P30*	F26	*USH2A*	NM_206933	c.2296T > C	p.(Cys766Arg)	[16]		c.2296T > C	p.(Cys766Arg)	[16]
*P32*	F28	*USH2A*	NM_206933	c.11713C > T	p.(Arg3905Cys)	[17]		c.9959-1G > C	p.(?)	[18]
*P35*	F31	*PDE6B*	NM_000283	c.1798G > A	p.(Asp600Asn)	[7]		c.1798G > A	p.(Asp600Asn)	[7]
*P37*	F32	*NR2E3*	NM_014249	c.119-2A > C	p.(?)	[19]		c.119-2A > C	p.(?)	[19]
*P39*	F33	*USH2A*	NM_206933	c.2276G > T	p.(Cys759Phe)	[20]		c.3684T > A	p.(Cys1228 *)	[13]
*P47*	F41	*USH2A*	NM_206933	c.953A > G	p.(Tyr318Cys)	LOVD ^‡^		c.5776 + 1G > C	p.(?)	this study

^‡^ Leiden Open Variation Database.

**Table 3:** Pathogenicity predictions for the novel missense variants reported in this study.

Gene	RefSeq	Nucleotide	Protein	In Silico Pathogenicity Analysis			
				MutationTaster ^†^	PolyPhen-2 ^‡^	SIFT *	Cadd13 ^#^
*CEP290*	NM_025114	c.1664A > T	p.(Lys555Ile)	LP	LP	P	27.8
*CEP290*	NM_025114	c.1092T > G	p.(Ile364Met)	LP	P	P	25.3
*PRPF31*	NM_015629	c.3G > A	p.(Met1Ile)	LP	B	P	23.8
*RHO*	NM_000539	c.560G > T	p.(Cys187Phe)	LP	P	P	24.8
*USH2A*	NM_206933	c.497A > G	p.(Glu166Gly)	LP	P	P	26.2
*USH2A*	NM_206933	c.3045C > G	p.(His1015Gln)	LP	LP	P	22.8
*USH2A*	NM_206933	c.6992G > A	p.(Gly2331Glu)	P	LP	P	28.9

^†^ http://www.mutationtaster.org/, ^‡^ Polymorphism Phenotyping v2; http://genetics.bwh.harvard.edu/pph2/, * Sorting Intolerant from Tolerant; http://sift.bii.a-star.edu.sg/, ^#^ Combined Annotation-Dependent Depletion; http://cadd.gs.washington.edu/, Abbreviations: B, benign; LP, likely pathogenic; P, pathogenic.

## Supplementary Materials

The following are available online at https://www.mdpi.com/1422-0067/21/1/86/s1, Table S1: Detailed clinical description of the pericentral RP cohort.

## Abbreviations

BCVA	Best corrected visual acuity
ERG	Electroretinogram
HGMD	Human Gene Mutation Database
LOVD	Leiden Open Variation Database
NGS	Next generation sequencing
OCT	Optical coherence tomography
RP	Retinitis pigmentosa

## References

[1] Hartong D.T., Berson E.L., Dryja T.P. (2006). Retinitis pigmentosa. Lancet.

[2] Wright A.F., Chakarova C.F., Abd El-Aziz M.M., Bhattacharya S.S. (2010). Photoreceptor degeneration: Genetic and mechanistic dissection of a complex trait. Nat. Rev. Genet..

[3] Sandberg M.A., Gaudio A.R., Berson E.L. (2005). Disease course of patients with pericentral retinitis pigmentosa. Am. J. Ophthalmol..

[4] Comander J., Weigel-DiFranco C., Maher M., Place E., Wan A., Harper S., Sandberg M.A., Navarro-Gomez D., Pierce E.A. (2017). The Genetic Basis of Pericentral Retinitis Pigmentosa-A Form of Mild Retinitis Pigmentosa. Genes (Basel).

[5] Matsui R., Cideciyan A.V., Schwartz S.B., Sumaroka A., Roman A.J., Swider M., Huang W.C., Sheplock R., Jacobson S.G. (2015). Molecular heterogeneity within the clinical diagnosis of pericentral retinal degeneration. Investig. Ophthalmol. Vis. Sci..

[6] Neveling K., Collin R.W., Gilissen C., van Huet R.A., Visser L., Kwint M.P., Gijsen S.J., Zonneveld M.N., Wieskamp N., de Ligt J. (2012). Next-generation genetic testing for retinitis pigmentosa. Hum. Mutat.

[7] Tsang S.H., Tsui I., Chou C.L., Zernant J., Haamer E., Iranmanesh R., Tosi J., Allikmets R. (2008). A novel mutation and phenotypes in phosphodiesterase 6 deficiency. Am. J. Ophthalmol..

[8] Shevach E., Ali M., Mizrahi-Meissonnier L., McKibbin M., El-Asrag M., Watson C.M., Inglehearn C.F., Ben-Yosef T., Blumenfeld A., Jalas C. (2015). Association between missense mutations in the BBS2 gene and nonsyndromic retinitis pigmentosa. JAMA Ophthalmol..

[9] Baux D., Blanchet C., Hamel C., Meunier I., Larrieu L., Faugere V., Vache C., Castorina P., Puech B., Bonneau D. (2014). Enrichment of LOVD-USHbases with 152 USH2A genotypes defines an extensive mutational spectrum and highlights missense hotspots. Hum. Mutat..

[10] Herrera W., Aleman T.S., Cideciyan A.V., Roman A.J., Banin E., Ben-Yosef T., Gardner L.M., Sumaroka A., Windsor E.A., Schwartz S.B. (2008). Retinal disease in Usher syndrome III caused by mutations in the clarin-1 gene. Investig. Ophthalmol. Vis. Sci..

[11] Pennings R.J., Te Brinke H., Weston M.D., Claassen A., Orten D.J., Weekamp H., Van Aarem A., Huygen P.L., Deutman A.F., Hoefsloot L.H. (2004). USH2A mutation analysis in 70 Dutch families with Usher syndrome type II. Hum. Mutat..

[12] Coppieters F., Casteels I., Meire F., De Jaegere S., Hooghe S., van Regemorter N., Van Esch H., Matuleviciene A., Nunes L., Meersschaut V. (2010). Genetic screening of LCA in Belgium: predominance of CEP290 and identification of potential modifier alleles in AHI1 of CEP290-related phenotypes. Hum. Mutat..

[13] Bonnet C., Riahi Z., Chantot-Bastaraud S., Smagghe L., Letexier M., Marcaillou C., Lefevre G.M., Hardelin J.P., El-Amraoui A., Singh-Estivalet A. (2016). An innovative strategy for the molecular diagnosis of Usher syndrome identifies causal biallelic mutations in 93% of European patients. Eur. J. Hum. Genet..

[14] Aller E., Jaijo T., Beneyto M., Najera C., Oltra S., Ayuso C., Baiget M., Carballo M., Antinolo G., Valverde D. (2006). Identification of 14 novel mutations in the long isoform of USH2A in Spanish patients with Usher syndrome type II. J. Med. Genet..

[15] Rosewich H., Ohlenbusch A., Gartner J. (2005). Genetic and clinical aspects of Zellweger spectrum patients with PEX1 mutations. J. Med. Genet..

[16] Glockle N., Kohl S., Mohr J., Scheurenbrand T., Sprecher A., Weisschuh N., Bernd A., Rudolph G., Schubach M., Poloschek C. (2014). Panel-based next generation sequencing as a reliable and efficient technique to detect mutations in unselected patients with retinal dystrophies. Eur. J. Hum. Genet..

[17] Krawitz P.M., Schiska D., Kruger U., Appelt S., Heinrich V., Parkhomchuk D., Timmermann B., Millan J.M., Robinson P.N., Mundlos S. (2014). Screening for single nucleotide variants, small indels and exon deletions with a next-generation sequencing based gene panel approach for Usher syndrome. Mol. Genet. Genomic Med..

[18] Sodi A., Mariottini A., Passerini I., Murro V., Tachyla I., Bianchi B., Menchini U., Torricelli F. (2014). MYO7A and USH2A gene sequence variants in Italian patients with Usher syndrome. Mol. Vis..

[19] Haider N.B., Jacobson S.G., Cideciyan A.V., Swiderski R., Streb L.M., Searby C., Beck G., Hockey R., Hanna D.B., Gorman S. (2000). Mutation of a nuclear receptor gene, NR2E3, causes enhanced S cone syndrome, a disorder of retinal cell fate. Nat. Genet..

[20] Rivolta C., Sweklo E.A., Berson E.L., Dryja T.P. (2000). Missense mutation in the USH2A gene: association with recessive retinitis pigmentosa without hearing loss. Am. J. Hum. Genet..

[21] Testa F., Rossi S., Colucci R., Gallo B., Di Iorio V., della Corte M., Azzolini C., Melillo P., Simonelli F. (2014). Macular abnormalities in Italian patients with retinitis pigmentosa. Br. J. Ophthalmol..

[22] Grondahl J., Riise R., Heiberg A., Leren T., Christoffersen T., Bragadottir R. (2007). Autosomal dominant retinitis pigmentosa in Norway: A 20-year clinical follow-up study with molecular genetic analysis. Two novel rhodopsin mutations: 1003delG and I179F. Acta Ophthalmol. Scand..

[23] Selmer K.K., Grondahl J., Riise R., Brandal K., Braaten O., Bragadottir R., Undlien D.E. (2010). Autosomal dominant pericentral retinal dystrophy caused by a novel missense mutation in the TOPORS gene. Acta Ophthalmol..

[24] Berson E.L., Rosner B., Weigel-DiFranco C., Dryja T.P., Sandberg M.A. (2002). Disease progression in patients with dominant retinitis pigmentosa and rhodopsin mutations. Investig. Ophthalmol. Vis. Sci..

[25] Haer-Wigman L., Newman H., Leibu R., Bax N.M., Baris H.N., Rizel L., Banin E., Massarweh A., Roosing S., Lefeber D.J. (2015). Non-syndromic retinitis pigmentosa due to mutations in the mucopolysaccharidosis type IIIC gene, heparan-alpha-glucosaminide N-acetyltransferase (HGSNAT). Hum. Mol. Genet..

[26] Sandberg M.A., Rosner B., Weigel-DiFranco C., McGee T.L., Dryja T.P., Berson E.L. (2008). Disease Course in Patients with Autosomal Recessive Retinitis Pigmentosa due to the USH2A Gene. Investig. Ophthalmol. Vis. Sci..

[27] Grover S., Fishman G.A., Alexander K.R., Anderson R.J., Derlacki D.J. (1996). Visual acuity impairment in patients with retinitis pigmentosa. Ophthalmology.

[28] Grover S., Fishman G.A., Anderson R.J., Tozatti M.S., Heckenlively J.R., Weleber R.G., Edwards A.O., Brown J. (1999). Visual acuity impairment in patients with retinitis pigmentosa at age 45 years or older. Ophthalmology.

[29] Berson E.L., Sandberg M.A., Rosner B., Birch D.G., Hanson A.H. (1985). Natural course of retinitis pigmentosa over a three-year interval. Am. J. Ophthalmol..

[30] Berson E.L., Rosner B., Sandberg M.A., Hayes K.C., Nicholson B.W., Weigel-DiFranco C., Willett W. (1993). A randomized trial of vitamin A and vitamin E supplementation for retinitis pigmentosa. Arch. Ophthalmol..

[31] Sandberg M.A., Rosner B., Weigel-DiFranco C., Dryja T.P., Berson E.L. (2007). Disease Course of Patients with X-linked Retinitis Pigmentosa due to RPGR Gene Mutations. Investig. Ophthalmol. Vis. Sci..

[32] Popovic P., Jarc-Vidmar M., Hawlina M. (2005). Abnormal fundus autofluorescence in relation to retinal function in patients with retinitis pigmentosa. Graefes Arch. Clin. Exp. Ophthalmol..

[33] Sujirakul T., Lin M.K., Duong J., Wei Y., Lopez-Pintado S., Tsang S.H. (2015). Multimodal Imaging of Central Retinal Disease Progression in a 2-Year Mean Follow-up of Retinitis Pigmentosa. Am. J. Ophthalmol..

[34] Burnight E.R., Wiley L.A., Drack A.V., Braun T.A., Anfinson K.R., Kaalberg E.E., Halder J.A., Affatigato L.M., Mullins R.F., Stone E.M. (2014). CEP290 gene transfer rescues Leber congenital amaurosis cellular phenotype. Gene Ther..

[35] Chacon-Camacho O.F., Zenteno J.C. (2015). Review and update on the molecular basis of Leber congenital amaurosis. World J. Clin. Cases.

[36] Nair A.A., Marmor M.F. (2017). ERG and other discriminators between advanced hydroxychloroquine retinopathy and retinitis pigmentosa. Doc. Ophthalmol..

[37] Iannaccone A., Kritchevsky S.B., Ciccarelli M.L., Tedesco S.A., Macaluso C., Kimberling W.J., Somes G.W. (2004). Kinetics of visual field loss in Usher syndrome Type II. Investig. Ophthalmol. Vis. Sci..

[38] Aboshiha J., Dubis A.M., Cowing J., Fahy R.T., Sundaram V., Bainbridge J.W., Ali R.R., Dubra A., Nardini M., Webster A.R. (2014). A prospective longitudinal study of retinal structure and function in achromatopsia. Investig. Ophthalmol. Vis. Sci..

[39] Fakin A., Jarc-Vidmar M., Glavac D., Bonnet C., Petit C., Hawlina M. (2012). Fundus autofluorescence and optical coherence tomography in relation to visual function in Usher syndrome type 1 and 2. Vis. Res..

[40] Musacchia F., Ciolfi A., Mutarelli M., Bruselles A., Castello R., Pinelli M., Basu S., Banfi S., Casari G., Tartaglia M. (2018). VarGenius executes cohort-level DNA-seq variant calling and annotation and allows to manage the resulting data through a PostgreSQL database. BMC Bioinform..

[41] Di Iorio V., Karali M., Brunetti-Pierri R., Filippelli M., Di Fruscio G., Pizzo M., Mutarelli M., Nigro V., Testa F., Banfi S. (2017). Clinical and Genetic Evaluation of a Cohort of Pediatric Patients with Severe Inherited Retinal Dystrophies. Genes (Basel).

[42] Schwarz J.M., Cooper D.N., Schuelke M., Seelow D. (2014). MutationTaster2: Mutation prediction for the deep-sequencing age. Nat. Methods.

[43] Adzhubei I., Jordan D.M., Sunyaev S.R. (2013). Predicting functional effect of human missense mutations using PolyPhen-2. Curr. Protoc. Hum. Genet..

[44] Kumar P., Henikoff S., Ng P.C. (2009). Predicting the effects of coding non-synonymous variants on protein function using the SIFT algorithm. Nat. Protoc..

[45] Rentzsch P., Witten D., Cooper G.M., Shendure J., Kircher M. (2019). CADD: Predicting the deleteriousness of variants throughout the human genome. Nucleic Acids Res..

